# Innovative graph neural network approach for predicting soil heavy metal pollution in the Pearl River Basin, China

**DOI:** 10.1038/s41598-024-67175-7

**Published:** 2024-07-17

**Authors:** Yannan Zha, Yao Yang

**Affiliations:** 1 Guangzhou Institute of Technology, Guangzhou, Computer Simulation Research and Development Center, 465 Huanshi East Road, Guangzhou, 510075 China; 2 Guangdong Provincial Key Laboratory of Agricultural & Rural Pollution Abatement and Environmental Safety, College of Natural Resources and Environment, Joint Institute for Environment & Education, South China Agricultural University, 483 Wushan St., Guangzhou, 510642 China; 3 Key Laboratory of Arable Land Conservation (South China), Ministry of Agriculture, Guangzhou, 510642 China

**Keywords:** Heavy metal distribution prediction, Graph neural network, Soil pollution, Geographic information, Environmental sciences, Natural hazards, Solid Earth sciences, Risk factors

## Abstract

Predicting soil heavy metal (HM) content is crucial for monitoring soil quality and ensuring ecological health. However, existing methods often neglect the spatial dependency of data. To address this gap, our study introduces a novel graph neural network (GNN) model, Multi-Scale Attention-based Graph Neural Network for Heavy Metal Prediction (MSA-GNN-HMP). The model integrates multi-scale graph convolutional network (MS-GCN) and attention-based GNN (AGNN) to capture spatial relationships. Using surface soil samples from the Pearl River Basin, we evaluate the MSA-GNN-HMP model against four other models. The experimental results show that the MSA-GNN-HMP model has the best predictive performance for Cd and Pb, with a coefficient of determination (R^2^) of 0.841 for Cd and 0.886 for Pb, and the lowest mean absolute error (MAE) of 0.403 mg kg^−1^ for Cd and 0.670 mg kg^−1^ for Pb, as well as the lowest root mean square error (RMSE) of 0.563 mg kg^−1^for Cd and 0.898 mg kg^−1^ for Pb. In feature importance analysis, latitude and longitude emerged as key factors influencing the heavy metal content. The spatial distribution prediction trend of heavy metal elements by different prediction methods is basically consistent, with the high-value areas of Cd and Pb respectively distributed in the northwest and northeast of the basin center. However, the MSA-GNN-HMP model demonstrates superior detail representation in spatial prediction. MSA-GNN-HMP model has excellent spatial information representation capabilities and can more accurately predict heavy metal content and spatial distribution, providing a new theoretical basis for monitoring, assessing, and managing soil pollution.

## Introduction

Soil serves as the cradle of life, providing the fundamental sustenance and development platform for all living organisms. However, with the influence of human activities and natural processes, the issue of heavy metal contamination in soils has become increasingly critical. Heavy metals, non-biodegradable elements, pose potential long-term negative impacts on the environment and human health due to their accumulation in soils. Therefore, it is of significant importance to predict and analyze the distribution of heavy metals in soils, as well as the factors influencing it, for both environmental protection and human health.

The original content of heavy metals in soil is primarily influenced by natural factors such as parent material, climate, biological activity, and topography^1^. However, with the rapid development of industrialization and urbanization, human activities (including industrial and agricultural pollution, transportation pollution, and household waste pollution) have become significant factors affecting the content of heavy metals in soil^2^. This has led to the complex spatial heterogeneity of soil heavy metals, making the prediction of their spatial distribution more difficult^3^. Classic statistical methods can only describe the overall variability of soil heavy metals content and fail to illustrate their spatial distribution characteristics^4^. Geostatistical models, by studying the spatial distribution and variability of variables with certain randomness and structure through variogram, have been widely used in predicting the spatial distribution of soil heavy metals^5^. However, these models have smoothing effects and struggle to identify local outliers of soil heavy metals; they also fail to describe the impact of environmental variables on the spatial differentiation of soil heavy metals^6^. With the development of 3S(Sensing, System and Service) technology^7^, environmental variables such as topography, climate, remote sensing, land use, and socio-economic factors have become more accessible, and methods like multiple linear regression, regression Kriging, and geographically weighted regression have been extensively used in regional soil heavy metal spatial distribution predictions^8–10^. However, such methods struggle to reveal the complex nonlinear relationships between soil heavy metals and environmental variables^11^.

Machine learning models such as artificial neural networks^12–15^, support vector machines^16^ and random forests^17,18^ have gradually been used for soil heavy metal spatial prediction. Folorunso et al. reviewed research predicting soil quality based on machine learning and analyzed the composition and quality of the soil, the prediction of soil parameters, existing soil datasets, soil maps, the influence of soil nutrients on crop growth, and the status of soil information system research^19^. Yang et al. proposed a new optimal sampling algorithm capable of realistically enhancing insufficient soil properties using machine learning uncertainty prediction^20^. Sun et al. developed a coupled retrieval approach to quantify nickel (Ni) concentration in agricultural soil using spaceborne hyperspectral imagery. Incorporating machine learning, multi-scale discrete wavelet transform, and other techniques, their work serves as a robust reference for agricultural applications worldwide^21^. Li et al. introduced an adaptive weighted normalization coupled with a linear weighted network framework for detecting chromium in soils^22^. Yang et al. developed machine learning models to study the adsorption of six heavy metals in soil, addressing the inefficiencies of traditional methods^23^. Pyo et al. focused on leveraging visible and near-infrared spectroscopy (VNIRS) combined with deep learning to estimate heavy metal concentrations in soil^24^. A convolutional neural network (CNN) was utilized to estimate arsenic, copper, and lead concentrations, showing higher accuracy compared to artificial neural network (ANN) and random forest regression (RFR) models.

Despite existing machine learning models having certain advantages in dealing with non-linear issues, they face challenges when dealing with high-dimensional data and complex network structures^25^. The spatial heterogeneity of soil heavy metal distribution is manifested in two aspects: (1) spatial dependence, which suggests that nearby locations might have similar soil heavy metal content due to possibly similar environmental conditions and human activities^26^, and (2) spatial self-organization, indicating that soil heavy metal distribution might demonstrate specific spatial patterns mainly due to self-organizing mechanisms in soil formation and development^27^. Current research employing machine learning models often assumes data are independent and identically distributed ^28^, overlooking the spatial dependence and self-organization of spatial data. Moreover, there is a notable gap in studies focused on regional pollutant risk predictions^29–31^. Graph Neural Networks (GNN) ^32,33^ are capable of directly handling network-structured data and capturing and utilizing information about spatial dependence and self-organization by learning the features of nodes and edges. Furthermore, GNNs can handle high-dimensional data and potentially reveal complex spatial patterns of soil heavy metal distribution through automatically learning data's inherent structure and patterns^34^. Therefore, GNNs offer a new possibility for handling the spatial heterogeneity of soil heavy metal distribution and complex geographical network structures.

The Pearl River Basin (PRB), a vital economic hub in southern China, faces significant environmental challenges due to rapid urbanization and diverse industrial activities. This region is marked by notable heavy metal contamination, a consequence of complex pollution pathways and varying parent material types. The PRB’s diverse industrial landscape and complex geology make it an ideal case study for understanding the intricate relationships influencing heavy metal distribution in soil. Accurate prediction of heavy metal distribution is crucial for environmental protection and human health, yet the spatial heterogeneity and complex interplay of factors have posed a considerable challenge. This study will utilize GNN to predict and analyze the distribution of heavy metals in the soil of the Pearl River Basin. By considering a variety of influencing factors, including geographical location, parent material type, pollution pathways, and types, we aim to accurately predict the distribution of heavy metals in the soil and analyze the main factors affecting it. The results of this study can contribute to a better understanding of the distribution patterns of heavy metals in soil and the main influencing factors, which hold significant importance for environmental protection and human health. At the same time, this study is the first to apply Graph Neural Networks to the prediction of heavy metal distribution, providing new insights and methods for further research on such issues.

The main contributions of this study are as follows.

1. Innovative Model Structure: We introduce an advanced MSA-GNN-HMP model that seamlessly integrates two pivotal components: MS-GCN and AGNN. Specifically, the MS-GCN component adeptly identifies multi-scale structural information within the graph, while the AGNN dynamically allocates weights to the neighboring nodes of each node. Experimental results validate the superior performance of our proposed model.
2. Deep Representation of Geographical Information: Our model offers a profound representation of geographical data, such as latitude and longitude, which is especially vital in predicting environmental issues like heavy metal concentrations.
3. In-depth Interpretation of Factors Affecting Soil Pb and Cd Concentrations: This research employs a data-driven approach to thoroughly analyze the various factors influencing Pb and Cd concentrations, providing mechanistic explanations that lay a solid theoretical foundation for soil heavy metal prevention.

## Materials

### Overview of the research area

**Figure 1:**
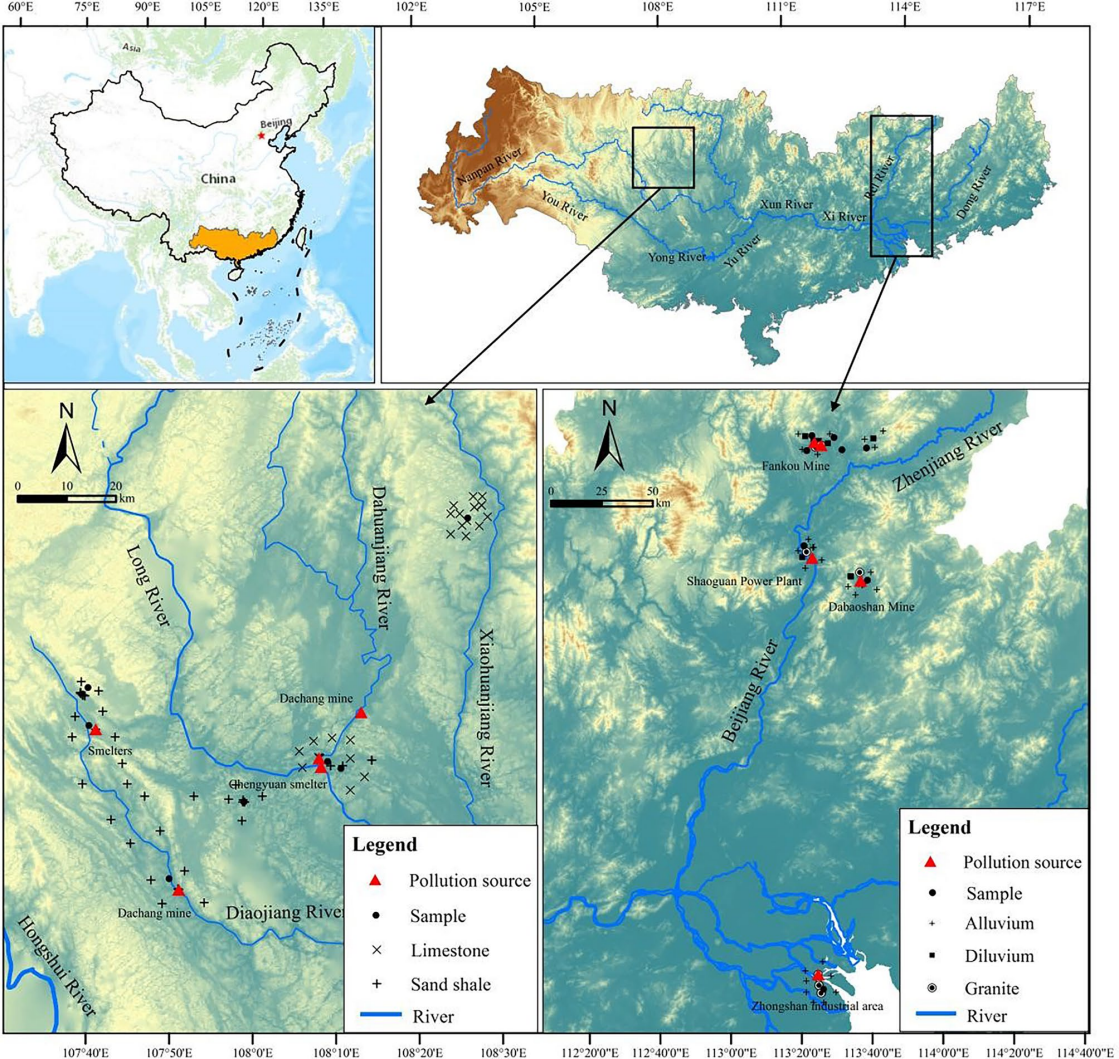
Schematic of pollution source and surrounding parent material and sample point distribution. Map generated using ArcGIS software (version 10.2). Geographic data was sourced from our field data collection (coordinates provided in the supplementary data file) and elevation information obtained from the National Geospatial Information website.

This study collected 142 surface soil samples from the Pearl River Basin, a region strategically selected due to its significance as a mining zone, and the sampling locations are depicted in Fig. 1. Additionally, the southern regions of China, particularly the Pearl River Basin, have been historically impacted by serious heavy metal contamination^35^ making it a critical area for such research^36^. These soil samples are spread across five different types of parent material developed soils, including limestone, sandy shale, alluvial deposits, diluvial deposits, and granite^37^. These soil samples come from different degrees of pollution and geological background regions. For this study, the primary contaminants of interest are cadmium (Cd) and lead (Pb). The choice to focus on these metals is based on their well-documented toxicity, which is among the highest for heavy metals^38^. Cadmium and lead can pose serious health risks to both humans and the environment, and their presence in the soil has the potential to be bioaccumulated in crops, thus entering the human food chain^39^.

The quantity of the samples collected is determined based on the size of the farmland area in different survey regions, which includes 18 samples of limestone developed soil, 36 samples of sandy shale developed soil, 42 samples of alluvial developed soil, 22 samples of diluvial developed soil, and 24 samples of granite developed soil. Each region selected multiple subregions including geological background areas, polluted areas, and mildly polluted areas. There are multiple sampling units and sampling points within each subregion. Two soil samples were collected from each sampling point, and each sample is a mixed sample of five soil samples. In addition, 6 unpolluted soil samples were collected from the same type of parent material developed soil region more than 10 km away from the pollution source. All samples were immediately transported to the laboratory after collection, a portion of which was used for microbial biomass carbon and nitrogen analysis, and the other part was used to determine basic soil physicochemical properties and total heavy metals content.

### Determination of basic soil properties and description of predictive variables

Soil pH was determined using a 2.5:1 soil water extract and pH meter; soil CEC was measured by the ammonium acetate exchange method^40^; soil texture was determined by the siphon method. Soil total organic matter (SOC) content was determined using the wet oxidation-potassium dichromate oxidation method; soil amorphous iron (FeTamm), amorphous manganese (MnTamm), crystalline iron (FeDCB), and crystalline manganese (MnDCB) content was determined by the o-phenanthroline colorimetric method; Dissolved organic matter (DOM) in the soil was extracted with water (soil to water ratio = 5:1) and oscillated for 1 h. The DOC content was determined using a TOC analyzer (vario TOC, Elementar, Germany). The DOM content of the soil was measured using a UV–visible spectrophotometer (UV-2450, Shimadzu, Japan). The absorbance values at 250, 254, 365, 400, 436, 465, 600, and 665 nm were measured. The specific UV–visible photometer value at 254 nm (SUVA254, L·g-1·cm-1) and ΔlogK were calculated according to Eqs. (1) and (2) ^41^.

1 $$ SUVA_{254} = A_{254} /lDOC $$

2 $$ \Delta \log K = \log A400 - \log A600 $$

$A_{i}$ is the absorbance value of DOM at i nm, and DOC is the soluble organic carbon content in the soil solution (mg L^−1^). SUVA254 and ΔlogK usually indicate the degree of humification of aromaticity and DOM^42^. E2/E3, E2/E4, and E4/E6 represent the absorbance ratios at 250 nm and 365 nm, 254 nm and 436 nm, and 465 nm and 665 nm, respectively. The first two ratios reflect DOM, the third ratio reflects the degree of humification, and shows the aromatic DOM^43^. Total heavy metals in the soil (Cd, Pb) were digested using the HCl-HNO3-HClO4-HF method^44^. The digest was measured using a flame atomic absorption spectrometer (Hitachi Z-2300, Hitachi, Japan). Each soil sample was set up in triplicate, and a blank test was also conducted at the same time.

According to the data sampling and measurement conditions, this paper selects a total of 15 variables(features) such as latitude and longitude as shown in the Table 1. Different geographical locations may have different climatic conditions, soil types, and human activities, and geographical location may affect the distribution of heavy metals; different types of pollution are the direct causes of heavy metal distribution and content; crops grown may affect the distribution of heavy metals in the soil, because different crops may have different absorption and accumulation capabilities for heavy metals in the soil; soil chemical properties such as organic matter content, pH value, iron and manganese content may affect the form and mobility of heavy metals in the soil, thereby affecting their distribution in the soil; the physical properties of the soil will affect the adsorption capacity for heavy metals, thereby affecting their distribution in the soil^45^. Based on the above 15 variables, this paper will predict the content of the two heavy metals Cd and Pb.

**Table 1:** Soil heavy metal prediction variables.

Category	Variables
Geographic information	Latitude, longitude
Pollution information	Type of pollution
Agricultural information	Crops grown
Soil chemical properties	Organic matter (g/kg), DOC (mg/kg), pH, free iron content (g/kg), amorphous iron content (g/kg), free manganese content (mg/kg), amorphous manganese content (mg/kg), CEC (cmol/kg)
Soil physical properties	Sand percentage, clay percentage, silt percentage

## Methods

The exploration of the spatiotemporal correlations at various monitoring points plays a critical role in enhancing the accuracy of heavy metal distribution predictions. This spatiotemporal correlation stems mainly from the distribution of geographic locations and the influences of factors like parent materials, pollution pathways, and types on each monitoring point. Graph structured data is the optimal solution for modeling the correlation between the spatial distribution of each monitoring point and the distribution of heavy metals. Nodes in the graph are used to represent monitoring points, and edges represent the correlation between the points. On the foundation of constructing a graph structure for heavy metal distribution, this article uses a graph neural network to realize heavy metal distribution prediction.

### Heavy metal distribution graph structure modeling

Under the influence of soil physicochemical properties and factors such as parent materials and pollution types, there exists a certain spatiotemporal correlation between monitoring points. This article models these monitoring points as graph-structured data, using nodes to represent each monitoring point and establishing connections between nodes to represent the potential correlation between the monitoring points.

Establishing connections between monitoring points is an essential part of constructing graph-structured data for heavy metal distribution. Unlike graph data such as social networks and transportation networks, which have clear node connection relationships, the construction of connection relationships between nodes within heavy metal distribution monitoring points relies on certain rules. Accurate node connection relationships are vital for effectively extracting features of graph signals. This section proposes different heavy metal distribution graph structure modeling methods under two scenarios: considering parent material distribution and not considering parent material distribution. These methods are used for subsequent prediction models.

#### Heavy metal distribution graph structure modeling considering geographic spatial distribution

Since the node connection relationships of most graph convolutional neural networks do not change with time, this article first outlines a method for modeling the graph structure of heavy metal distribution without considering parent material distribution.

When the spatial distribution of parent materials is not taken into account, the geographic location of a monitoring point is the primary determinant of its similarity in heavy metal distribution. Factors such as soil type, environmental temperature, and atmospheric conditions may differ significantly at different locations at the same time, thereby affecting the correlation of heavy metal distribution between monitoring points. Monitoring points that are closer together have a stronger correlation in heavy metal distribution due to similar environmental conditions and minor latitude and longitude differences; conversely, monitoring points farther apart have weaker correlations.

Therefore, this paper proposes a fixed graph structure modeling method that takes into account the distance between monitoring points. For any monitoring point, the number of its neighbor points is determined based on the distance from other points to this monitoring point. By establishing connection relationships between each monitoring point and its neighboring points, a graph structure for heavy metal distribution is ultimately formed.

For any two monitoring points *v*_*i*_ and *v*_*j*_ within the study area, let *d*_*ij*_ represent the distance between nodes *v*_*i*_ and *v*_*j*_, and *M*_nb,i_ represents the number of neighboring points for *v*_*i*_. Firstly, the distances from all the monitoring points in the study area to *v*_*i*_ are calculated, as follows:

3 $$ \{ d_{ij} \left| {v_{j} \in V,v_{j} \ne v_{i} } \right.\} $$

The number of neighboring points is a key parameter that determines the density of the adjacency matrix^32^. To consider the correlation between different monitoring points' heavy metal concentration under the premise of ensuring the sparsity of the adjacency matrix, this paper calculates the distance of related monitoring points through the Haversine formula based on longitude and latitude and further defines the number of neighboring nodes *M*_nb,i_ as follows:

4 $$ g_{i} (x) = Card\left( {v_{j} \left| {d_{ij} \le d} \right.} \right) $$

5 $$ M_{nb,i} = g_{i} (d) $$

That is, for each node *v*_*i*_, the number of neighboring nodes is the number of monitoring points within a distance d kilometers from it.

After obtaining the number of neighboring nodes *M*_nb,i_, the closest *M*_nb,i_ nodes to node *v*_*i*_ are chosen to form the neighboring node set $N(v_{i} )$. The connection relationship is established between each node *v*_*i*_ and its neighboring point $v_{j} ,v_{j} \in N\left( {v_{i} } \right)$, i.e., $e_{ij} \in E$. If *A* is the preliminary adjacency matrix, then:

6 $$ A(i,j) = \left\{ \begin{gathered} 1\quad if\;v_{j} \in N(v_{i} ) \hfill \\ 0\quad else \hfill \\ \end{gathered} \right. $$

After completing the above steps, several connected components of the cluster graph structure are obtained. This paper uses the reachable matrix of the undirected graph to judge the graph's connectivity and realize the connection of connected components. If *A* is the adjacency matrix of graph *G*, one of the methods to calculate the reachable matrix $M_{G}$ is as follows:

Let $A_{i} = {\text{sgn}}_{M} \left( {\left( {A + In} \right)^{i} } \right)$, calculate *A*_1_, *A*_2_,… in sequence. If $A_{k - 2} \ne A_{k - 1} = A_{k}$, then $M_{G} = A_{k}$. The function ${\text{sgn}} M(X)$ applies the sign function to all elements of matrix X and outputs a homomorphic matrix *Y*, i.e.

7 $$ Y(i,j) = {\text{sgn}} \left( {X\left( {i,j} \right)} \right) $$

The rank of the reachable matrix *rank*(*M*_*G*_) is the number of connected components. Each vector in the largest linearly independent group of this matrix corresponds to the nodes contained in each non-connected subgraph.

**Figure 2:**
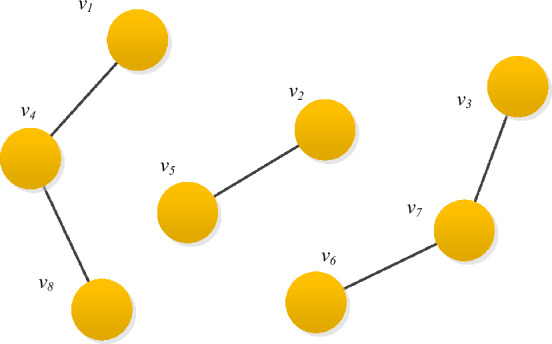
Example of a non-connected graph.

Taking the graph structure shown in Fig. 2 as an example, this graph contains 3 connected components. The calculated result of its reachable matrix is as follows:

8 $$ M_{G} = \left( {\begin{array}{*{20}c} 1 & 0 & 0 & 1 & 0 & 0 & 0 & 1 \\ 0 & 1 & 0 & 0 & 1 & 0 & 0 & 0 \\ 0 & 0 & 1 & 0 & 0 & 1 & 1 & 0 \\ 1 & 0 & 0 & 1 & 0 & 0 & 0 & 1 \\ 0 & 1 & 0 & 0 & 1 & 0 & 0 & 0 \\ 0 & 0 & 1 & 0 & 0 & 1 & 1 & 0 \\ 0 & 0 & 1 & 0 & 0 & 1 & 1 & 0 \\ 1 & 0 & 0 & 1 & 0 & 0 & 0 & 1 \\ \end{array} } \right) $$

In this matrix, *rank*(*M*_*G*_) = 3, and the three vectors $$x1 = [1,0,0,1,0,0,0,1]^{T}$$, $$x2 = [0,1,0,0,1,0,0,0]^{T}$$, and $$x3 = [0,0,1,0,0,1,1,0]^{T}[0,0,1,0,0,1,1,0]$$$$^{T}$$ make up the maximum linearly independent group of this matrix, $$M_{G}$$, and correspond to the three connected components, with node sets $$V_{1} = \{ v_{1} ,v_{4} ,v_{8} \}$$,$$V_{2} = \{ v_{2} ,v_{5} \}$$, $$V_{3} = \{ v_{3} ,v_{6} ,v_{7} \}$$.

After identifying the number of connected components and the nodes they contain using the reachable matrix, we connect the nodes that are the shortest distance apart between non-connected components to ensure the connectivity of the entire regional cluster graph structure, and update the edge set E. Based on this, the weighted adjacency matrix of the fixed cluster graph structure, $A_{w}$, can be obtained. Its calculation method is as follows:

9 $$ A_{w} \left( {i,j} \right) = \left\{ \begin{gathered} \exp \left( { - \frac{{\left( {d_{ij} } \right)^{2} }}{{2\theta^{2} }}} \right),\quad if\;e_{ij} \in E \hfill \\ 0,\quad \quad \quad \quad \quad \quad otherwise \hfill \\ \end{gathered} \right. $$

**Figure 3:**
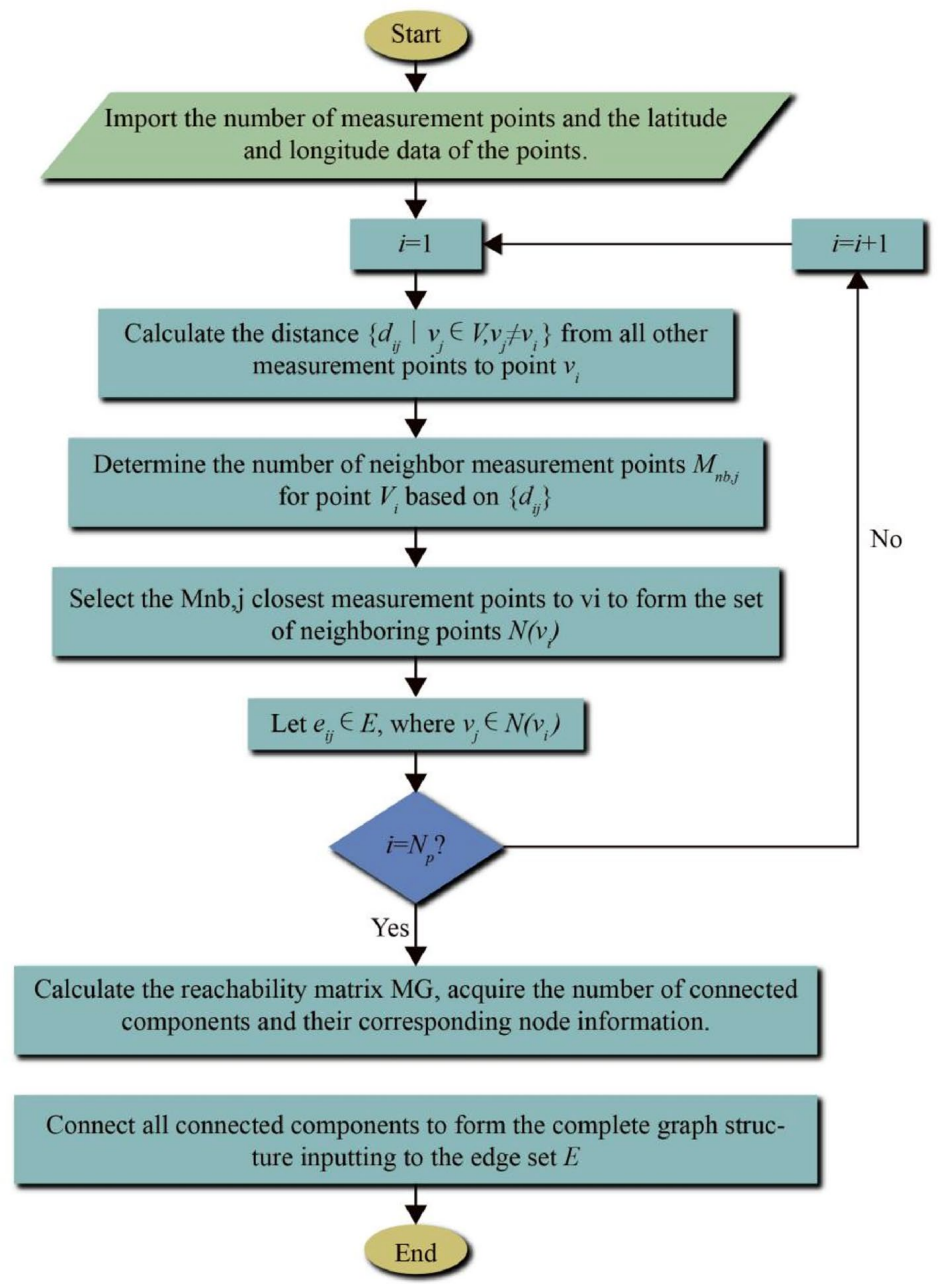
Modeling process of fixed graph structure for measurement point clustering.

In this paper, we set *θ* = 5. Figure 3 shows the modeling process of the fixed cluster graph structure based on the measurement point distance proposed in this paper.

#### Parent material clustering analysis and sampling site environmental type classification

Soil parent material is the foundation for soil formation. Different soil parent materials can lead to different heavy metal contents and distributions within the soil, thereby leading to some correlations in heavy metal distribution across sampling sites. For these heavy metal distribution sampling sites, constructing an appropriate graph structure based on parent material distribution and geographic location of the sites holds significant value for accurately extracting temporal and spatial correlations of heavy metal distribution. Given the vast geographical range and complex parent material distribution of heavy metal distribution sampling sites in the Pearl River Basin, the characteristics of heavy metal distribution under different parent materials can vary. Therefore, this paper first uses a clustering algorithm to classify the parent material, then determines the environmental type by assessing the coverage of parent material at each site. This allows us to divide the entire heavy metal distribution into several subclusters, simplifying the process of graph structure modeling.

The clustering algorithm is one of the commonly used algorithms in data classification. Given the complexity of the parent material distribution, distance-based clustering algorithms, such as K-means, KNN, etc., may not be suitable for parent material area division. This paper chooses the DBSCAN clustering algorithm for parent material area division. This method does not require specifying the number of clusters beforehand, it has resistance to noise, it is insensitive to outliers, and can handle clusters of any shape and size.

In graph convolutional models, the connection relationships between nodes in the graph represent the transmission of information, and the features of connected nodes often tend to be consistent. For heavy metal distribution sampling sites, the characteristics of heavy metal distribution under different environmental conditions are varied. To differentiate these characteristics, we classify the environmental conditions at each site into three types based on the distribution of parent material around the site: organic-rich, mineral-rich, and mixed type.

For any given sampling site *v*_*i*_, we consider certain features of the site, such as location, latitude, longitude, and parent material. By analyzing these features, we classify the environmental conditions of the site. In this classification, we define an "Environmental Type Index (ETI)" that can be calculated based on the content of organic matter and mineral matter at the site. For example, if the organic content at a site is higher than a certain threshold, we classify it as "organic-rich"; if the mineral content is higher than a certain threshold, we classify it as "mineral-rich"; if both are not high, but both exist, we classify it as "mixed".

After determining the environmental status of each site within the cluster, we define the environmental type matrix *M*_*eit*_ for use in subsequent weighted adjacency matrix calculations. The matrix is defined as follows:

10 $$ M_{eit} (i,j) = \left\{ \begin{gathered} 1\;\;\;if\;environmental\;type\;of\;site\;v_{i} \;is\;the\;same\;as\;site\;v_{j} \hfill \\ 0\;\;\;otherwise \hfill \\ \end{gathered} \right. $$

#### Dynamic graph structure modeling of heavy metal distribution sampling sites considering parent material type

In the graph structure modeling of heavy metal distribution sampling sites considering parent material type, we first calculate the set of neighboring nodes for each sampling site based on geographic location in “Heavy metal distribution graph structure modeling considering geographic spatial distribution”, and form a preliminary adjacency matrix *A*. We then modify *A* based on the parent material type matrix from “Parent material clustering analysis and sampling site environmental type classification”, as follows:

11 $$ A^{\prime} = A \odot M_{eit} $$

where *A'* is the preliminary adjacency matrix considering parent material type, *A* is the preliminary adjacency matrix considering the geographic location of the sampling sites, *M*_*eit*_ is the parent material type matrix defined in “Parent material clustering analysis and sampling site environmental type classification”, and *⊙* represents the Hadamard product. The specific meaning of this modification method is: for any node *v*_*i*_ within the cluster, eliminate the nodes in the set of neighboring nodes *N(v*_*i*_*)* that have different parent material types from *v*_*i*_, and establish the connection relationship between vi and the remaining nodes to get *A'*.

Let the set of edges corresponding to matrix *A'* be *E'*. After calculating the reachable matrix using *A'*, we connect the nodes that are closest to each other between different connected components after judging the connectivity of the graph, update *E'*, and finally form the graph structure of heavy metal distribution sampling sites considering the parent material type. Let *A*_*w*_ be the corresponding weighted adjacency matrix, and its calculation method is as follows:

12 $$ A_{w} = W \odot M_{adj} $$

where $W \in R^{N \times N}$ is the distance weight coefficient of the sampling points. The spatial position of the sampling point does not change, but *W* may change because the connection relationship *E'* may change:

13 $$ W(i,j) = \left\{ \begin{gathered} \exp \left( { - \frac{{\left( {d_{ij} } \right)^{2} }}{{2\theta^{2} }}} \right),\quad if\,e_{ij} \in E^{\prime} \hfill \\ 0,\quad \quad \quad \quad \quad \quad otherwise \hfill \\ \end{gathered} \right. $$

**Figure 4:**
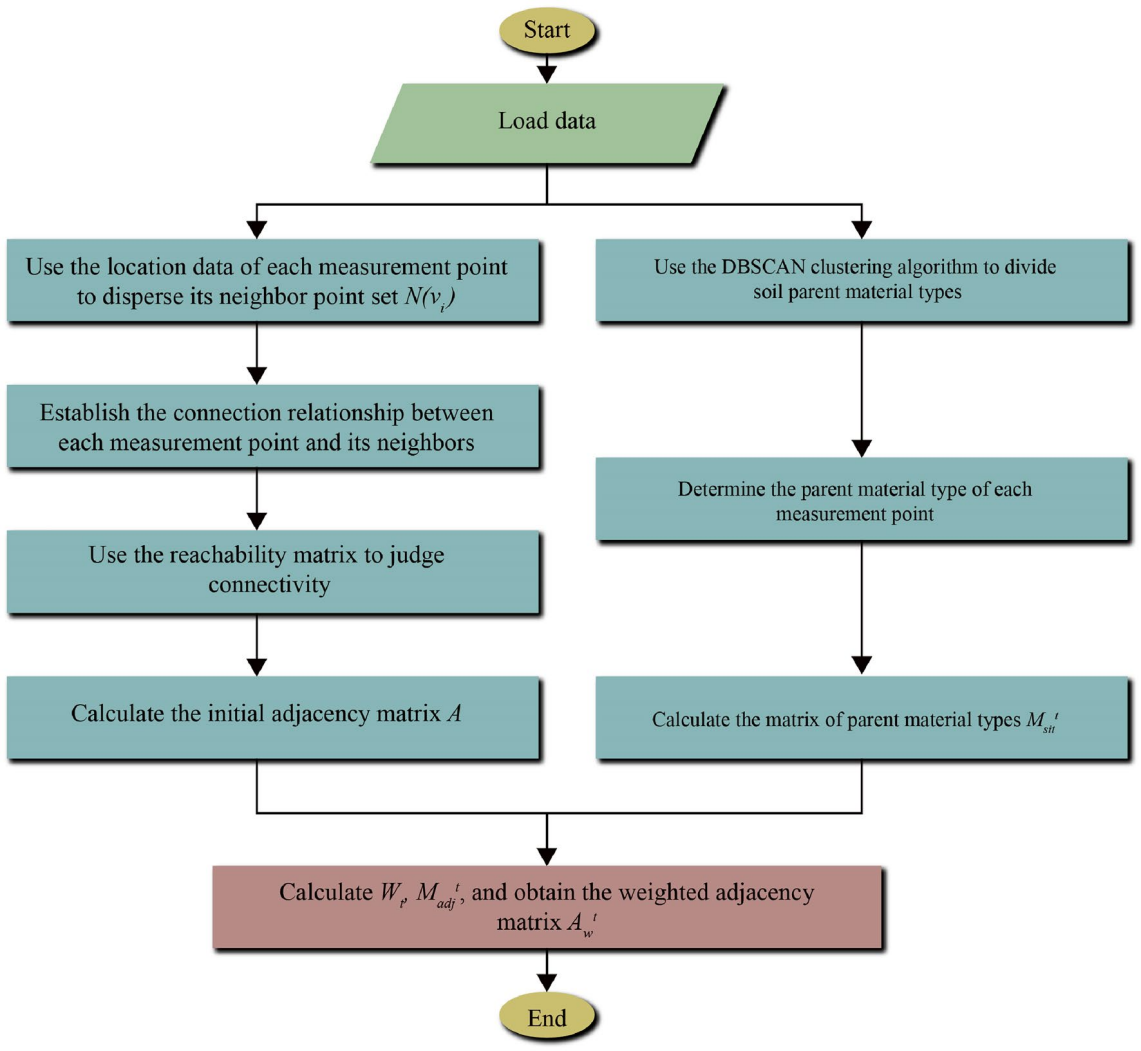
Modeling process of sampling site cluster graph structure considering parent material type.

Ee take *θ* = 5. Figure 4 shows the modeling process of the graph structure of the sampling site cluster considering the parent material type. By analyzing the parent material type of each sampling site, we can establish a corresponding graph structure and calculate the weighted adjacency matrix to generate the weighted adjacency matrix.

For future graph structure data, we first use the sampling site's parent material relative position prediction method based on the parametric equation to get the predicted position of each sampling site. Assume that the predicted position of sampling site *v*_*i*_ is (*x*_*i*_, *y*_*i*_), analyze the parent material type of sampling site iv based on the distribution of parent material around the sampling site, thereby obtaining the parent material type matrix *M*_*eit*_, and further calculate the weighted adjacency matrix *A*_*w*_.

Following these steps, using the predicted position of each sampling site, according to the partition results of parent material, a dynamically changing sequence of adjacency matrices *A*_1_, *A*_2_, …, *A*_n_ can be generated, achieving prediction of the graph structure.

### Heavy metal distribution prediction based on attention graph convolution model

Graph Convolutional Neural Networks can effectively process graph structured information. However, traditional GCNs usually assume that all neighbor nodes have the same impact on the target node when processing node features. Such an assumption might not be applicable in many real-world scenarios. For example, in the task of predicting soil heavy metal distribution, different neighbor nodes (i.e., different geographical locations) might have different impacts on the target node (i.e., target geographical location), which depends on various factors such as their geographical distance, soil type, etc.

In contrast, the Attention-based Graph Neural Network (AGNN) can dynamically allocate weights for each neighbor node of a target node, reflecting the importance of the neighbor nodes to the target node. This mechanism can better capture complex relationships between nodes and therefore improve the predictive performance of the model. In predicting soil heavy metal distribution, the AGNN can dynamically assign weights according to the degree of influence different neighbor nodes (i.e., different geographical locations) have on the target node (i.e., target geographical location), and thus more accurately predict the heavy metal distribution at the target location.

**Figure 5:**
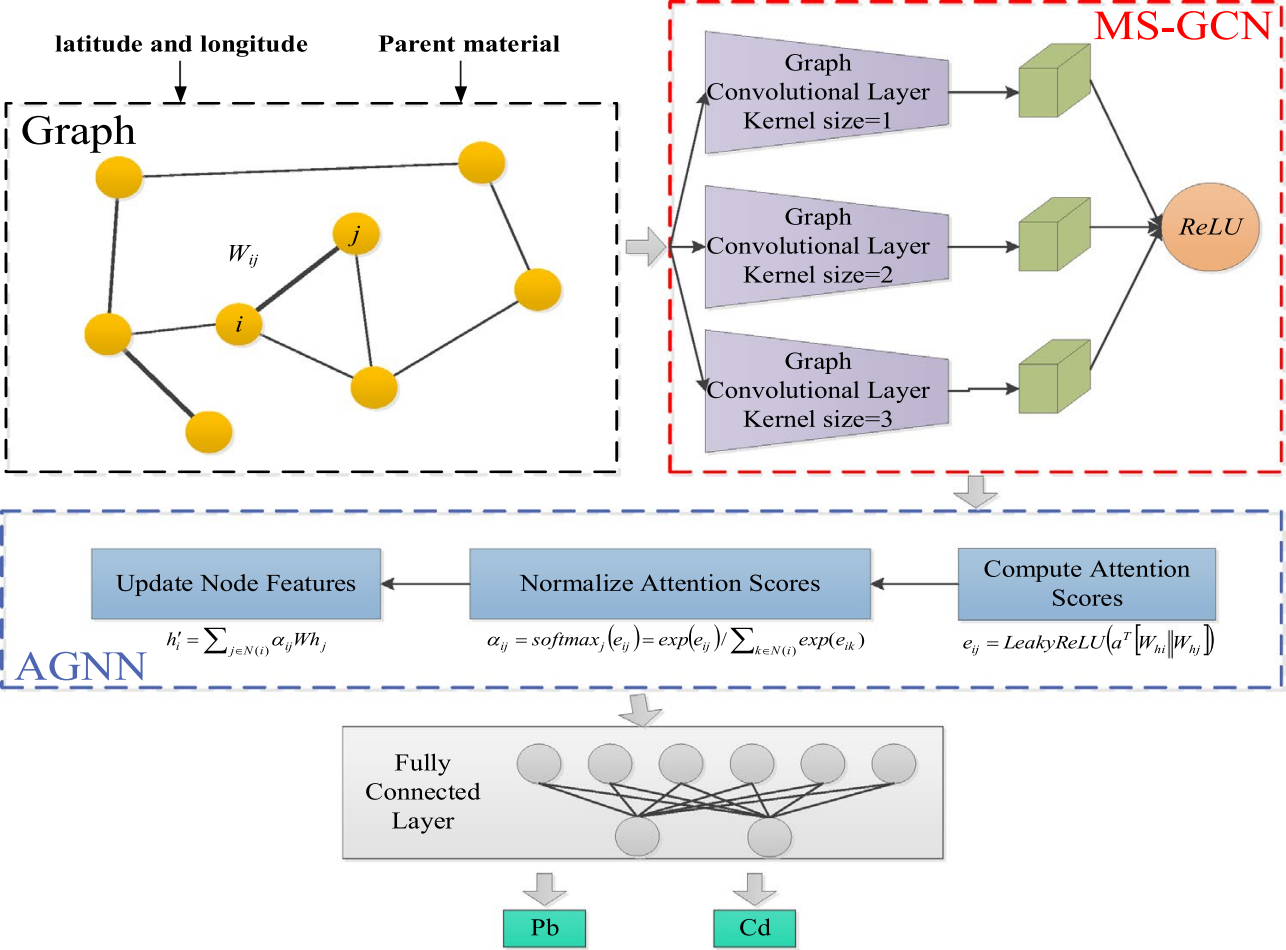
Architecture of the MSA-GNN-HMP model.

This paper proposes the heavy metal distribution prediction model as shown in Fig. 5. It should be noted that the graph depicted here is schematic, and the final figure can be found in Fig. S1. The model combines the Multi-Scale Graph Convolutional Network (MS-GCN) and Attention-based Graph Neural Network (AGNN). We refer to it as MSA-GNN-HMP (Multi-Scale Attention-based Graph Neural Network for Heavy Metal Prediction).

1. Multi-scale graph convolutional network (MS-GCN) module.
  In the Multi-Scale Graph Convolutional Network (MS-GCN), we use graph convolution operations to handle graph data. The graph convolution operation can be considered as a convolution operation on the structure of the graph, which can capture the local structural information of nodes in the graph. In MS-GCN, we have designed three parallel graph convolution layers with small, medium, and large receptive fields, respectively, to capture graph structural features at different scales.
  **Small receptive field graph convolution layer** It is mainly used to capture the local structural information in the graph. We can set the size of the convolution kernel to 1, i.e., only consider the features of the node itself and its direct neighbors. The computation formula for the graph convolution operation is:
  14 $$ H^{(1)} = \sigma \left( {D^{ - 1/2} AD^{ - 1/2} H^{(0)} W^{(1)} } \right) $$
  where *A* is the adjacency matrix of the graph, *D* is the degree matrix, *H*^(0)^ is the input node feature matrix, *W*^(1)^ is the weight matrix of the first graph convolution layer, and σ is the activation function.
  **Medium receptive field graph convolution layer** This layer is mainly used to capture medium-scale structural information in the graph. We can set the size of the convolution kernel to 2, i.e., consider the features of the node itself and its two-hop neighbors. The computation formula for the graph convolution operation is:
  15 $$ H^{(2)} = \sigma \left( {D^{ - 1/2} A^{2} D^{ - 1/2} H^{(0)} W^{(2)} } \right) $$
  where *A*^2^ is the square of the adjacency matrix of the graph, representing the connection relationship between two-hop neighbors, and the meanings of the other symbols are the same as above.
  **Large receptive field graph convolution layer** This layer is mainly used to capture global structural information in the graph. We can set the size of the convolution kernel to 3, i.e., consider the features of the node itself and its three-hop neighbors. The computation formula for the graph convolution operation is:
  16 $$ H^{(3)} = \sigma \left( {D^{ - 1/2} A^{3} D^{ - 1/2} H^{(0)} W^{(3)} } \right) $$
  where *A*^3^ is the cube of the adjacency matrix of the graph, representing the connection relationship between three-hop neighbors, and the meanings of the other symbols are the same as above.
  The outputs of these three graph convolution layers will be merged into one feature map, then passed through an activation function (like ReLU) for a non-linear transformation. In this way, the MS-GCN can capture structural information from the graph at different scales.
2. Attention-based graph neural network (AGNN).
  After MS-GCN, we designed an Attention-based Graph Neural Network (AGNN). In AGNN, we use a graph attention mechanism to assign weights to each neighbor node of a node, reflecting the importance of neighbor nodes to the target node. Specifically, AGNN can be implemented using the following steps:
  **Compute attention scores** For each node in the graph, first calculate the attention scores with its neighbor nodes. The attention scores are computed based on the features of nodes and can be calculated using the following formula:
  17 $$ e_{ij} = {\text{LeakyReLU}}\left( {a^{T} \left[ {W_{hi} \left\| {W_{hj} } \right.} \right]} \right) $$
  where *hi* and *hj* are the features of node *i* and node *j*, respectively, *W* is the weight matrix, *a* is the parameter of the attention mechanism, || represents concatenation, and *LeakyReLU* is the activation function.
  **Normalize attention scores** In order to make the attention scores fall between 0 and 1, use the softmax function to normalize the attention scores:
  18 $$ \alpha_{ij} = {\text{softmax}}_{j} \left( {e_{ij} } \right) = {\text{exp}}\left( {e_{ij} } \right)/\sum\nolimits_{k \in N(i)} {{\text{exp}}(e_{ik} )} $$
  where *N*(*i*) represents the set of neighbor nodes of node *i*.
  **Update node features** Finally, use the normalized attention scores to assign weights to each neighbor node of a node, and then average the features of neighbor nodes by weight to obtain new node features:
  19 $$ h^{\prime}_{i} = \sum\nolimits_{j \in N(i)} {\alpha_{ij} Wh_{j} } $$
  AGNN can dynamically assign weights to each neighbor node of a node, reflecting the importance of neighbor nodes to the target node. This mechanism can enhance the graph neural network model's ability to represent geographical information (longitude and latitude).

### Evaluation metrics

In order to provide a rigorous evaluation of the predictive performance for the distribution of heavy metals in the soil, this study applies a nested cross-validation (CV) strategy alongside independent training/testing splits to ensure a robust and unbiased model assessment. Specifically, a k-fold cross-validation strategy, commonly with k = 10, is embedded within another layer of cross-validation to optimize model parameters while preventing data leakage between the training and test sets during the assessment phase.

Moreover, to further enhance the reliability of our model evaluation, we conduct 100 independent training/testing splits. For each split, the dataset is randomly partitioned into training and test sets, and the model is trained and validated accordingly. This iterative process assists in safeguarding against potential biases or anomalies that may emerge from a single random data split, thereby offering a more stable and reliable performance estimate.

Within each training/testing split or fold in the CV, the model is trained, and subsequently, the predicted values are compared with the actual measured values, with prediction accuracy being assessed by calculating the Mean Absolute Error (MAE), Root Mean Squared Error (RMSE), and Coefficient of Determination (*R*^2^) across all folds:

19 $$ MAE = \frac{1}{n}\sum\limits_{i = 1}^{n} {\left| {P_{i} - O_{i} } \right|} $$

20 $$ RMSE = \sqrt {\frac{1}{n}\sum\limits_{i = 1}^{n} {\left( {O_{i} - P_{i} } \right)^{2} } } $$

21 $$ R^{2} = {1 - }\frac{{\sum\nolimits_{i = 1}^{n} {\left( {P_{i} - \overline{O}_{i} } \right)^{2} } }}{{\sum\nolimits_{i = 1}^{n} {\left( {O_{i} - \overline{O}_{i} } \right)^{2} } }} $$

where *P*_i_ and *O*_i_ are the predicted and actual values of the *i*-th sample, respectively; $\overline{O}_{i}$ is the average of the actual values; and *n* is the number of samples in the test set.

## Experiments and analysis

### Experimental setup

The experiments were conducted on a custom-built workstation equipped with an Intel(R) Core(TM) i9-10900K CPU (10 cores) running at 3.70 GHz, an NVIDIA GeForce RTX 3080 GPU. The software environment was anchored on Ubuntu 20.04 LTS, utilizing Python 3.8.5. For data manipulation and numerical operations, The MSA-GNN-HMP model specifically being implemented in the Deep Graph Library (DGL). During the training process, Model training was initiated with parameters set at a batch size of 8, 100 epochs, and an Adam optimizer with a learning rate of 0.001. Figure S2 shows the training curve of the model proposed in this paper, and it can be seen that the model converges around epoch 80.

### Characteristics of heavy metal content in the soil of the Pearl River Basin

The descriptive statistics of the two soil heavy metals, Cd and Pb, in the study area are shown in Table S2. The average content of Cd and Pb are 1.58 mg kg^−1^ and 105.83 mg kg^−1^, respectively. The coefficient of variation can reflect the extent of the impact of environmental changes and human activities on the accumulation of heavy metals in the soil. In this data set, the coefficients of variation for Cd and Pb are 1.37% and 1.67%, respectively. This could indicate that the distribution of these two heavy metals in the soil of the Pearl River Basin is not uniform, and may be influenced by human activities to a certain extent. The skewness and kurtosis of the data can reflect the shape of the distribution. Here, the skewness of Cd and Pb are 4.16 and 2.92, respectively, both positively skewed, indicating that the content of both heavy metals is lower in most samples, but there are some samples with higher content, which may be due to severe pollution in some areas. At the same time, the kurtosis of Cd and Pb are 24.73 and 8.53, respectively, higher than the kurtosis of a normal distribution, indicating that the distribution of these two heavy metals is more concentrated around the average value, and there are more outliers.

**Figure 6:**
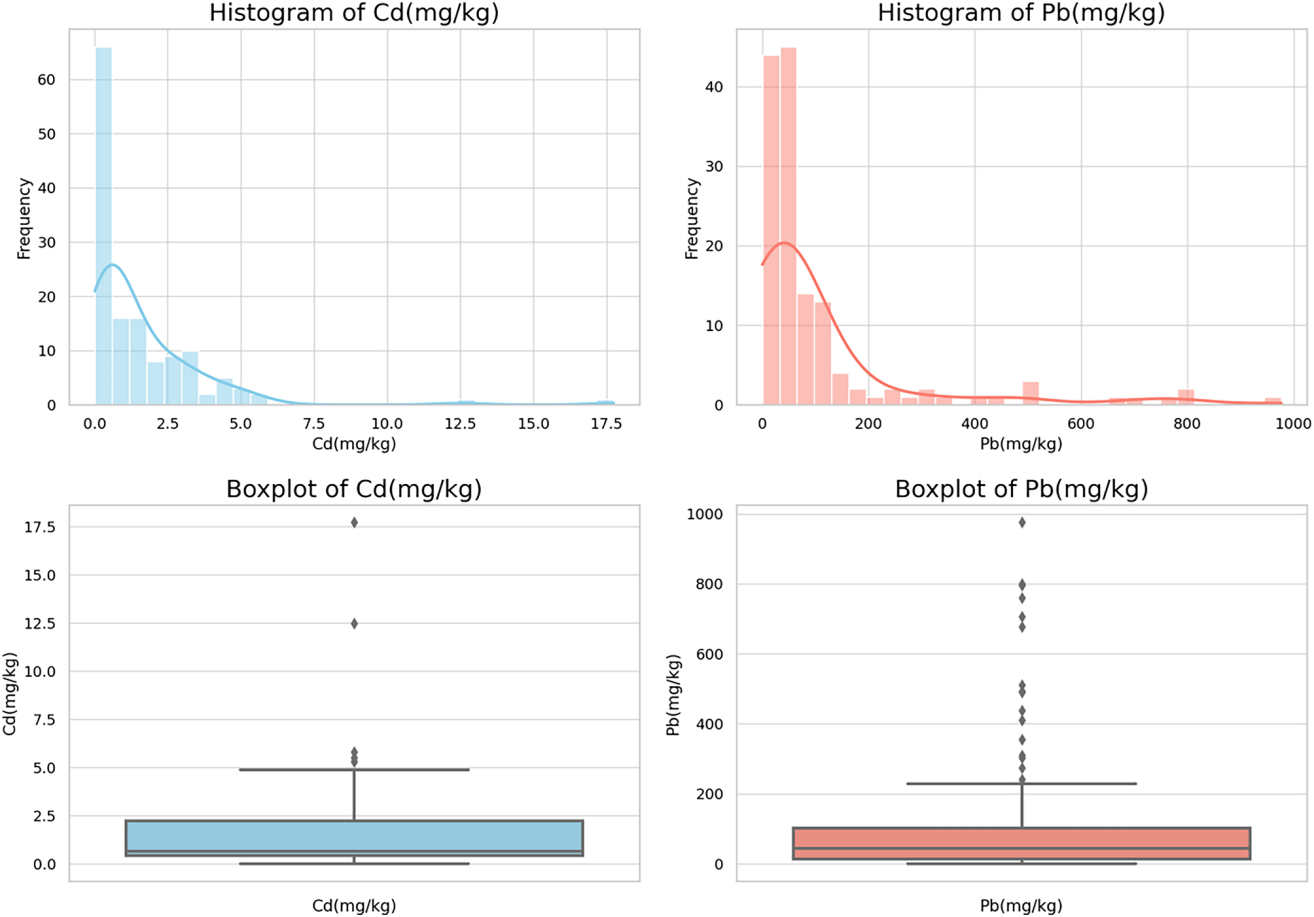
Descriptive statistics of Cd and Pb.

Figure 6 is the histogram and box plot of the two heavy metals. In the histogram and box plot for "Cd", we can see that the data for "Cd" concentration is right-skewed, with some larger values. At the same time, the box plot shows many outliers, which are all values greater than 1.5 times the interquartile range from the upper quartile. In the histogram and box plot for "Pb", we can also see that the data for "Pb" concentration is right-skewed, with some larger values. Similarly, the box plot shows many outliers, which are all values greater than 1.5 times the interquartile range from the upper quartile. Overall, the distribution of the two heavy metals in the study area is extremely uneven.

### Model prediction accuracy analysis

**Figure 7:**
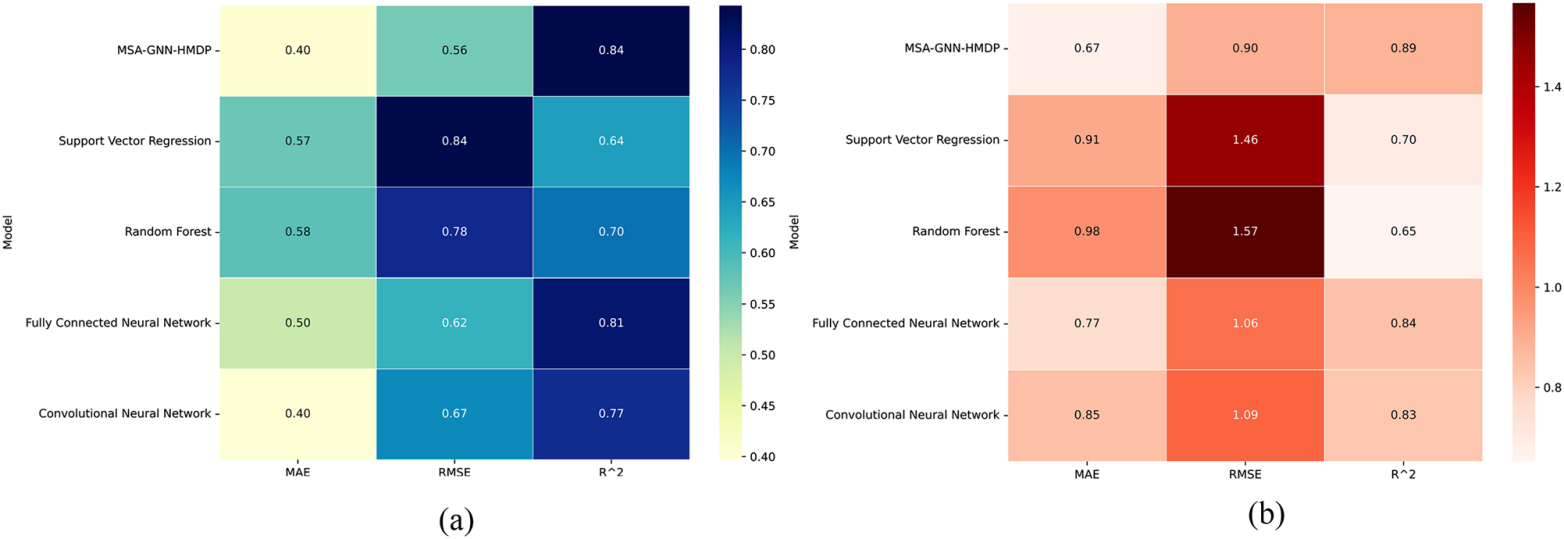
Model evaluation metrics heatmap for predicting Cd (**a**) and Pb (**b**).

We compared the MSA-GNN-HMP with four other models, namely Support Vector Regression, Random Forest, Fully Connected Neural Network, Convolutional Neural Network, and Spatial Random Forests^45^, on the test set. During the training process, the training hyperparameters for the aforementioned comparison models can be found in Table S1. The MAE, RMSE, and R^2^ values of each model are shown in Fig. 7.

We observe a distinct advantage of the MSA-GNN-HMP model over other models for soil heavy metal (Cd and Pb) prediction. A comprehensive examination of the metrics Mean Absolute Error (MAE), Root Mean Square Error (RMSE), and Coefficient of Determination (R^2^) further elucidates the superiority of the MSA-GNN-HMP model.

Concerning the MAE, a standard metric used to evaluate the prediction error, the MSA-GNN-HMP model demonstrated the smallest error in both Cd and Pb prediction. Specifically, the MSA-GNN-HMP model meticulously captures the spatial dependencies between different geographical locations through its attention-based Graph Neural Network (AGNN), enabling it to discern intricate patterns in the distribution of Cd and Pb. This nuanced understanding minimizes the prediction error, particularly when compared to models like Support Vector Regression, which might struggle with the spatial heterogeneity and non-linearity in the data.

Likewise, an analysis of the RMSE scores, another common gauge for prediction error, reveals that the MSA-GNN-HMP model surpasses other models in terms of accuracy for both Cd and Pb. It’s noteworthy that models like Random Forest and Fully Connected Neural Network, while being potent in various predictive tasks, might fall short in accurately mapping the spatial distribution due to their inability to inherently consider spatial relationships among data points, which is a crucial aspect in predicting heavy metal concentrations in soil.

Further reinforcing the model's supremacy, the R^2^ values, indicative of the accuracy of prediction, were highest for the MSA-GNN-HMP model for both Cd and Pb. This suggests that the model not only offers precision but also provides an exceptional fit to the observed data.

Spatial Random Forests (SRF) is a variant of the traditional Random Forests model, which incorporates spatial autocorrelation into the model by considering the spatial relationship between observations. Spatial Random Forests exhibited commendable predictive accuracy, particularly with an MAE and RMSE of 0.460, 0.710 for Cd, and 0.720, 0.960 for Pb, respectively. While the model does leverage spatial dependencies, which is a critical factor in predicting soil heavy metal distribution. However SRF performed worse compared to the MSA-GNN-HMP model.

The MSA-GNN-HMP model, leveraging Multi-Scale Graph Convolutional Networks (MS-GCN) and attention-based Graph Neural Networks (AGNN), effectively captures geographical information and the influence of neighboring nodes on the target node. This renders superior predictive results for soil heavy metal distribution, underscoring the importance of harnessing geographical information and considering spatial relationships when addressing such issues. Furthermore, this validates the potential of Graph Neural Networks for handling graph-structured geographical information.

### Ablation experiment

**Figure 8:**
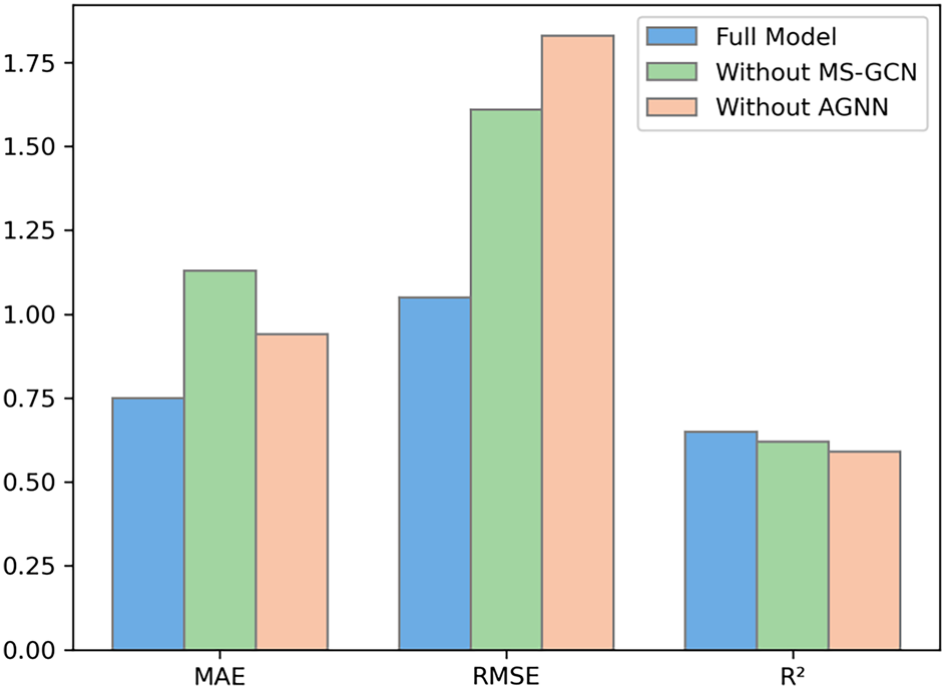
Ablation experiment results.

To validate the roles of the MS-GCN and AGNN parts in the MSA-GNN-HMP model, we conducted ablation experiments. We removed the MS-GCN and AGNN respectively and observed the changes in model performance. The experimental results are shown in Fig. 8.

Firstly, we ablated the MS-GCN part, i.e., we only retained the AGNN part. In this case, the model only considered the importance of each node to its neighboring nodes, without capturing the different scale structure information in the graph. We found that, compared to the complete MSA-GNN-HMP model, removing MS-GCN resulted in an increase in MAE to 1.13, an increase in RMSE to 1.61, and a decrease in R^2^ to 0.62. These results demonstrate that the MS-GCN part plays a crucial role in the model. It effectively captures different scale structure information in the graph, enhancing the predictive performance of the model.

Next, we ablated the AGNN part, i.e., we only retained the MS-GCN part. In this case, the model only considered the different scale structure information in the graph without weighting the importance of each node to its neighboring nodes. We found that, compared to the complete MSA-GNN-HMP model, removing AGNN increased the MAE to 0.94, RMSE to 1.83, and decreased R^2^ to 0.59. These results suggest that the AGNN part also plays a significant role in the model. It dynamically assigns weights to the neighboring nodes of each node, enhancing the model's representation ability for geographical information (latitude and longitude).

Through the ablation experiments, we can see that both the MS-GCN and AGNN parts play crucial roles in the MSA-GNN-HMP model, jointly improving the predictive performance of the model. This validates our original intention of designing the MSA-GNN-HMP model: to combine MS-GCN and AGNN, capturing different scale structure information in the graph, and dynamically assigning weights to the neighboring nodes of each node, to enhance the predictive accuracy of the model.

### Factor importance analysis

**Figure 9:**
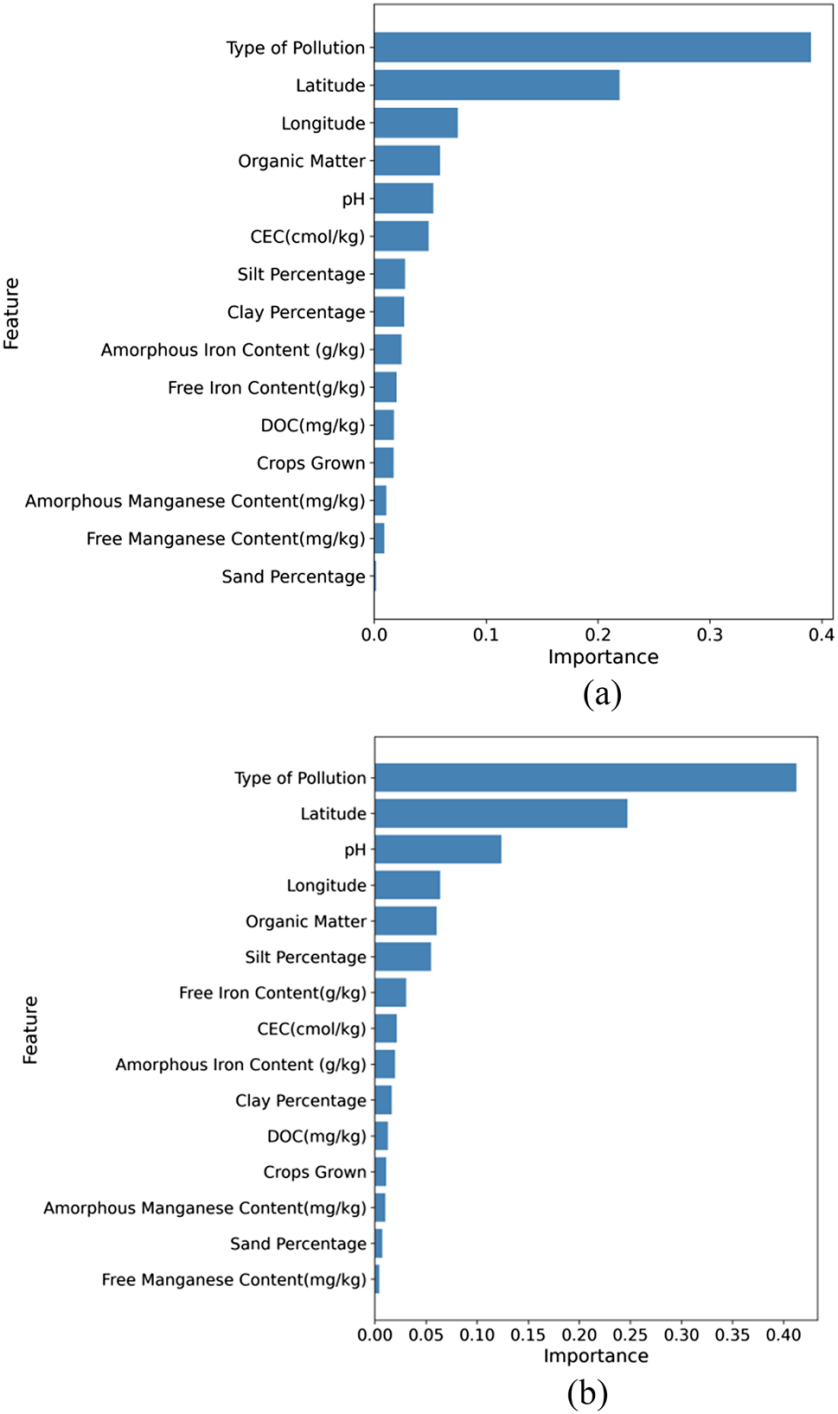
Feature importance analysis. (**a**) Feature Importance for Predicting Cd. (**b**) Feature Importance for Predicting Pb.

**Figure 10:**
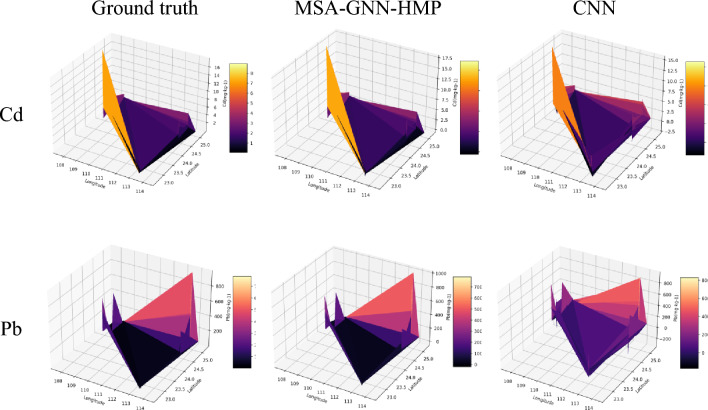
Comparison of spatial prediction of soil heavy metals in the Pearl River Basin by MSA-GNN-HMP and CNN.

We examined the significance of features in predicting heavy metal concentrations of Cd and Pb, as shown in Figs. 9 and 10. The Fig. S3a,b illustrate the correlation and fitting between the top six significant features and the content of Cd and Pb, respectively.Notably, for both Cd and Pb, "Type of Pollution" emerged as the most critical feature with weights of 0.3904 and 0.4126, respectively. This implies that the source of pollution plays a pivotal role in determining the levels of Cd and Pb in the soil. Latitude ranks second in feature importance for both metals, with weights of 0.2192 and 0.2472, indicating that geographic location also significantly influences the soil concentrations of Cd and Pb.

Latitude ranks second in feature importance for both metals, with weights of 0.2192 and 0.2472, indicating that geographic location also significantly influences the soil concentrations of Cd and Pb. In our study, latitude and longitude play a key role in reflecting the multiple factors influencing the distribution of heavy metals in soils. They serve as proxies for climatic conditions, soil properties, and human activities. For instance, climatic conditions affect soil pH and thus heavy metal availability, with these changes being reflected by different latitudes and longitudes^46,47^. The latitude and longitude in our graph neural network also indicate the distance from the Pearl River Basin, where heavy metal content varies with the migration and change of surface runoff. Heavy metals migrate laterally with surface runoff, expanding the contamination range and potentially reducing their content due to deposition^48^. In our study area, rivers flow from west to east and north to south, representing a "lateral migration process" from upstream to downstream, mirrored by changes in latitude and longitude. Incorporating latitude and longitude into our model comprehensively reflects influencing factors and accurately predicts heavy metal distribution, offering a new perspective on the complex mechanisms of soil heavy metal distribution.

Other features, such as Organic Matter, pH, and CEC, vary in their importance between the two metals but still exert noticeable impacts. Some features, like Sand Percentage, hold relatively lower importance for Cd (0.0016), yet they remain noteworthy.

### Analysis of heavy metal distribution prediction

As shown in Fig. 10 , the spatial distribution trends of the same soil heavy metal elements Pb and Cd predicted by the two different prediction methods, MSA-GNN-HMP and CNN, are largely consistent. In the predictions made by the MSA-GNN-HMP model, the high value areas of Cd are mainly distributed in the northwestern part of the sample area, while the high value areas of Pb are mainly distributed in the northeastern part. The MSA-GNN-HMP model is superior to the CNN model in predicting the low-value areas, reflecting more clearly the local variation of soil Pb and Cd content. The MSA-GNN-HMP model is better able to capture the microscopic distribution features of both heavy metals (Pb and Cd), and the prediction bias for high and low values is significantly reduced.

## Discussion

This study provides a detailed analysis of the distribution characteristics of the two heavy metals, Cd and Pb, in the soils of the Pearl River Basin, highlighting their uneven distribution and potential anthropogenic impacts. Statistical analysis shows that these two heavy metals have relatively low content in most samples, but there are some samples with high content, suggesting potential varying levels of soil contamination. Due to the harmfulness of these two heavy metals in the environment, such research is significant for understanding the degree of soil contamination and formulating effective remediation strategies.

In the comparison of prediction models, the MSA-GNN-HMP model demonstrated superior performance, outperforming other models in predictive accuracy and fitting results for soil heavy metal content. This is attributed to the roles of the MS-GCN and AGNN parts in the MSA-GNN-HMP model, with the MS-GCN part effectively capturing different scale structural information in the graph, and the AGNN part enhancing the model's representation ability for geographical information (latitude and longitude) through dynamic weight allocation. These results affirm the original intention and effectiveness of the MSA-GNN-HMP model design. Further ablation experiments show that both the MS-GCN and AGNN parts play important roles in the MSA-GNN-HMP model. Removal of either part leads to a decline in model performance, further affirming their importance within the model and validating the necessity of this model design.

Although our study provides an innovative model to predict soil heavy metal distribution, we acknowledge that there are certain uncertainties, particularly at finer spatial scales. The MSA-GNN-HMP model, while effective at a broader scale, may not fully capture the local variations in areas with intricate spatial features. Future research should integrate higher resolution data and additional spatial analysis methods to improve the model's precision and reliability ^49,50^.

Our findings indicate that latitude and longitude significantly influence soil heavy metal distribution. These effects are indirect, as latitude and longitude serve as proxies for a complex set of environmental variables, including climatic conditions, soil properties, and human activities. For instance, climatic conditions affect soil pH and thus the availability of heavy metals, with these changes being reflected by different latitudes and longitudes ^46^. The latitude and longitude in our graph neural network also indicate the distance from the Pearl River Basin, where heavy metal content varies with the migration and change of surface runoff. Heavy metals migrate laterally with surface runoff, expanding the contamination range and potentially reducing their content due to deposition ^48^. In our study area, rivers flow from west to east and north to south, representing a "lateral migration process" from upstream to downstream, mirrored by changes in latitude and longitude. Incorporating latitude and longitude into our model comprehensively reflects influencing factors and accurately predicts heavy metal distribution, offering a new perspective on the complex mechanisms of soil heavy metal distribution.

In our study, we closely examined the key factors affecting the levels of Cd and Pb in the soil. For Cd, the type of pollution stands out as the most influential factor. This suggests that some pollution sources, like specific industrial activities, are direct contributors of Cd to the environment. Latitude also plays an important role, influencing how organic materials in the soil break down. These organic materials can combine with Cd, potentially affecting how it behaves in the soil and its availability to plants. Longitude gives us insights into how different areas, with varied land uses and industrial activities, might have differing amounts of Cd in the soil. Organic matter, known for its ability to bind with metals, can interact with Cd, impacting its movement and availability in the soil. The behavior of Pb in soil shows some differences compared to Cd. The type of pollution remains a dominant factor for Pb levels, highlighting the role of external sources. Latitude's impact might be linked to specific soil characteristics and plant types in an area, which can influence the form and availability of Pb in the soil. The relationship between soil pH and Pb is well-known: a more acidic soil can increase Pb's solubility, making it easier for plants to absorb or for it to seep into groundwater. Finally, just like with Cd, longitude and organic matter also play roles in determining Pb levels in the soil.

In the spatial distribution map of heavy metal distribution prediction, we can see that the results predicted by the MSA-GNN-HMP model are fairly consistent with the actual distribution. Particularly in areas with low heavy metal content, the MSA-GNN-HMP model can more clearly reflect their local variations. This indicates that the MSA-GNN-HMP model is not only superior in prediction accuracy compared to other models but also more accurate in capturing the microscopic distribution features of soil heavy metals. Figure S4 demonstrates the application of our MSA-GNN-HMP model in conducting a probabilistic risk assessment for heavy metal contamination in the Pearl River Basin. This Monte Carlo simulation provides a risk-based perspective essential for guiding environmental restoration and management actions. It showcases the model’s capability in offering a spectrum of risk scenarios, enabling targeted interventions and strategic planning for environmental protection.

In summary, this study demonstrates that the MSA-GNN-HMP model is a highly effective tool for predicting soil heavy metal content, aiding us in better understanding the distribution of heavy metals in the soils of the Pearl River Basin.

## Conclusion

This study has delineated the distribution patterns of heavy metals, specifically cadmium (Cd) and lead (Pb), within the soil samples of the Pearl River Basin. The analysis revealed notable disparities in the distribution of these heavy metals, with average concentrations of 1.58 mg kg^−1^ for Cd and 105.83 mg kg^−1^ for Pb, suggesting an uneven distribution that could be attributed to human activities and accumulation.

Model prediction accuracy analysis showed that the MSA-GNN-HMP model performed well in predicting the spatial distribution of Cd and Pb in soil. The model had the lowest mean absolute error (MAE) and root mean square error (RMSE) and the highest coefficient of determination (R^2^), indicating its excellent ability to predict the distribution of these heavy metals.

In addition, the study also demonstrated that the MSA-GNN-HMP model integrates MS-GCN and AGNN components, which can effectively capture the spatial heterogeneity of heavy metal distributions.The MS-GCN module efficiently extracts the multi-scale structural information from the graph, which improves the prediction performance of the model, while the AGNN component dynamically assigns weights to neighbouring nodes, which enhances the model's ability to represent geographic information, such as latitude and longitude. capability. These findings confirm the design principle of the MSA-GNN-HMP model, which combines MS-GCN and AGNN to improve prediction accuracy.

This study has fundamentally revealed the distribution characteristics and potential risks of heavy metals in soils near heavy industrial areas in the Pearl River Basin. The results of the study indicate the urgency of taking preventive measures and provide a strong scientific basis for local governments to formulate targeted risk control and pollution management strategies.

### Supplementary Information

Supplementary Information.

## Data Availability

All data generated or analyzed during this study are included in this manuscript.
