# Supplementary material for: Innovative graph neural network approach for predicting soil heavy metal pollution in the Pearl River Basin, China
Source: Sci Rep. 2024 Jul 17;14:16505. doi: 10.1038/s41598-024-67175-7 (PMC11255285; doi:10.1038/s41598-024-67175-7)
Supplement: Supplementary file 1 — Supplementary Information. [file 41598_2024_67175_MOESM1_ESM.docx]

**Figures**

**S1：**

S1 displays the visualization of the graph neural network obtained after training, where the red dots represent the sample points, and the blue lines represent the association weights between these sample points.


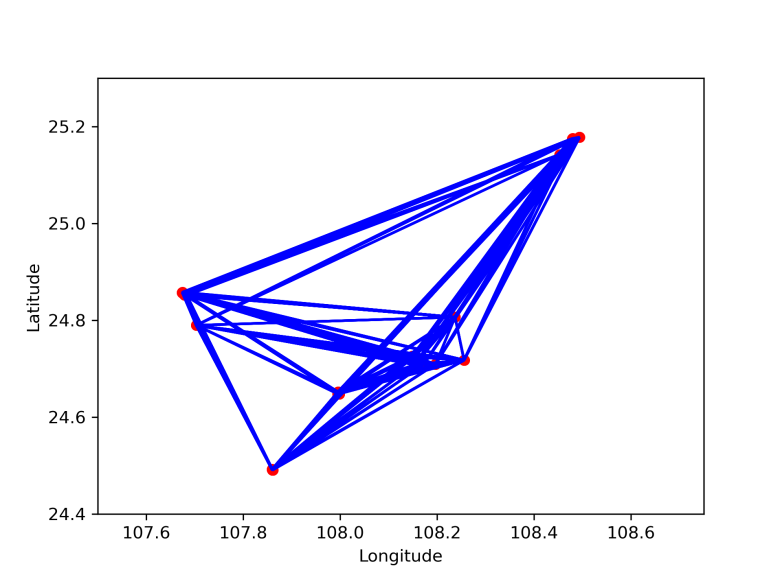


S1 Visualization of Nodes and Edges in Graph Neural Networks

**S2：**

Figure S2 shows the training curve of the model proposed in this paper, and it can be seen that the model converges around epoch 80.


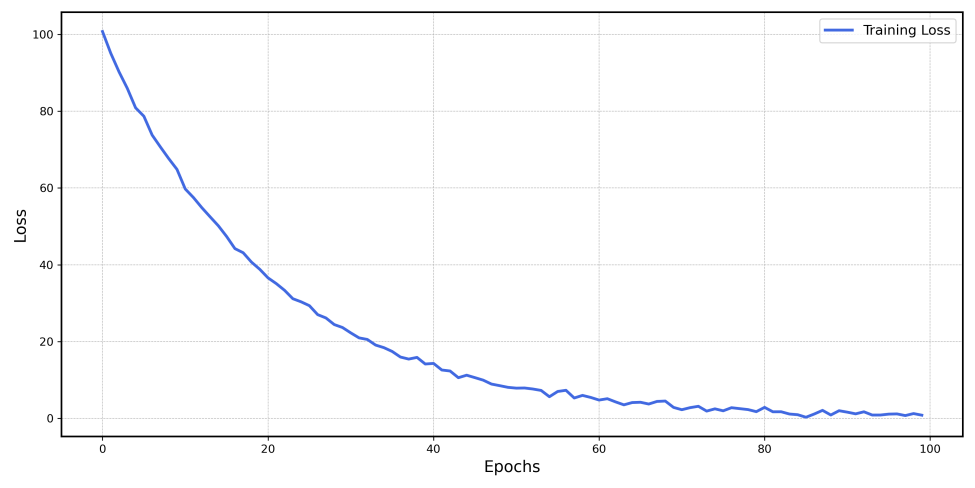


S2 Training Loss over Epochs

**S3：**

The figures S3(a) and S3(b) illustrate the correlation and fitting between the top six significant features and the content of heavy metals, specifically for Cd and Pb, respectively.


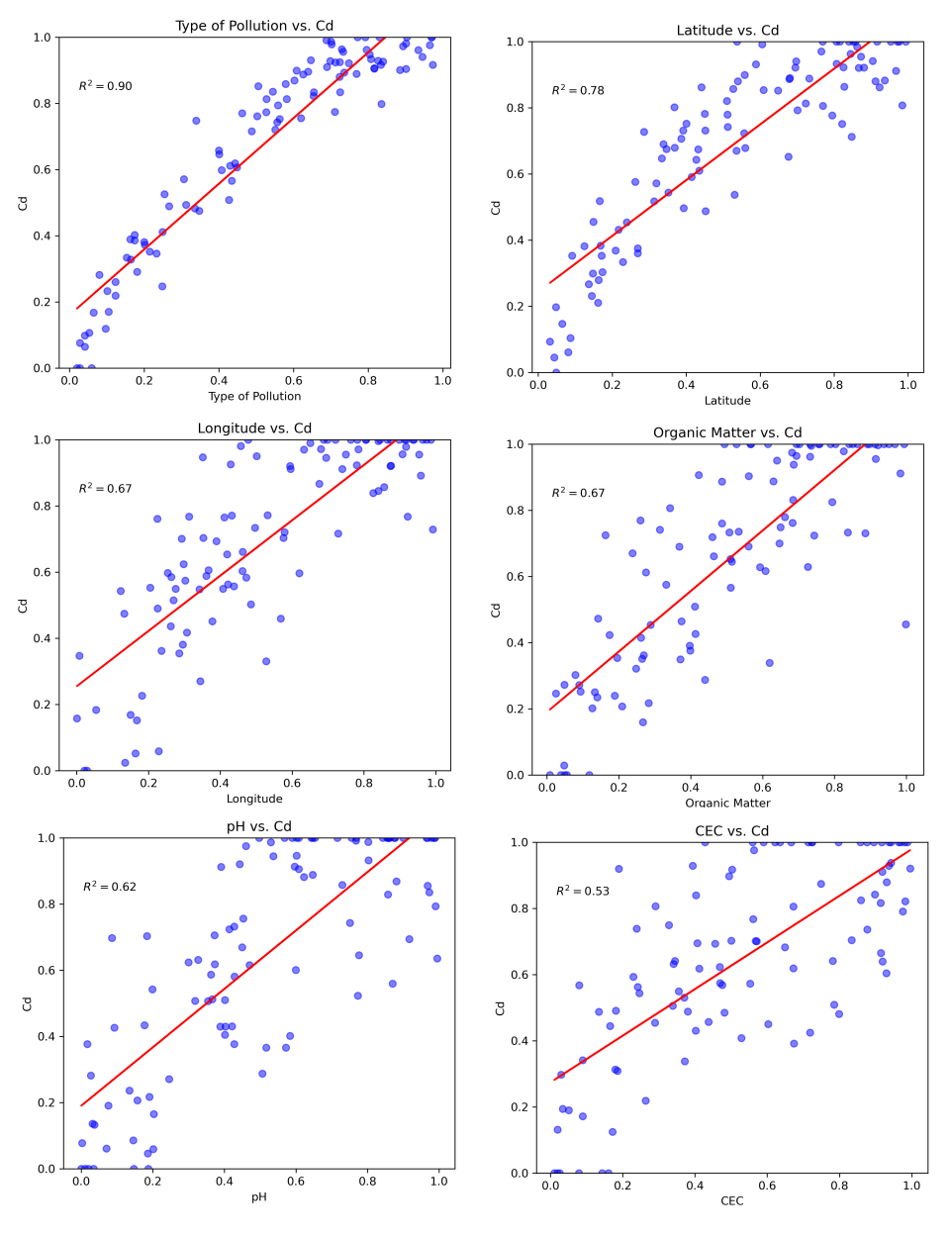


S3(a) Analysis of the relationship between the top 6 important features and Cd


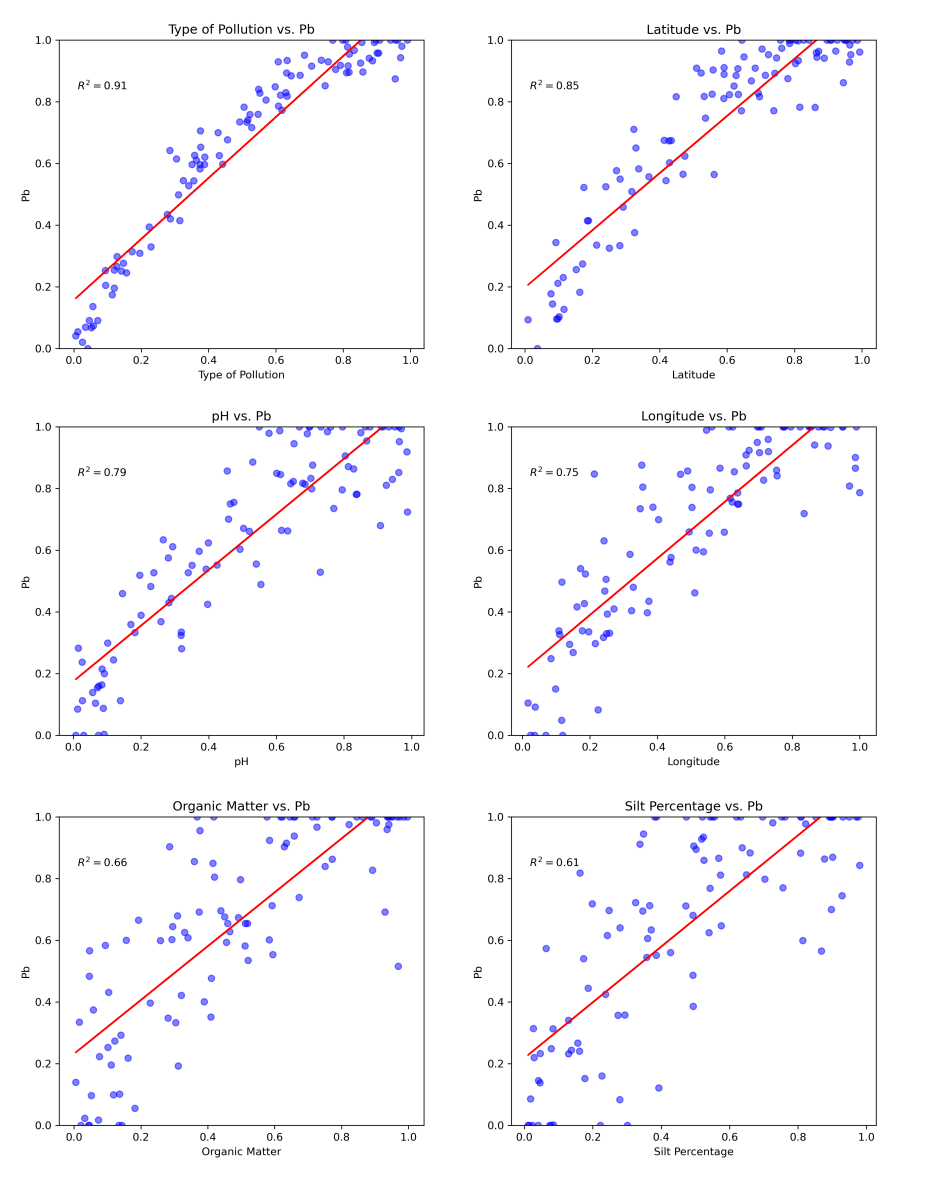


S3(b) Analysis of the relationship between the top 6 important features and Pb

S4： Monte Carlo simulation based on heavy metal data predicted by the MSA-GNN-HMP model is shown in Figure.S4


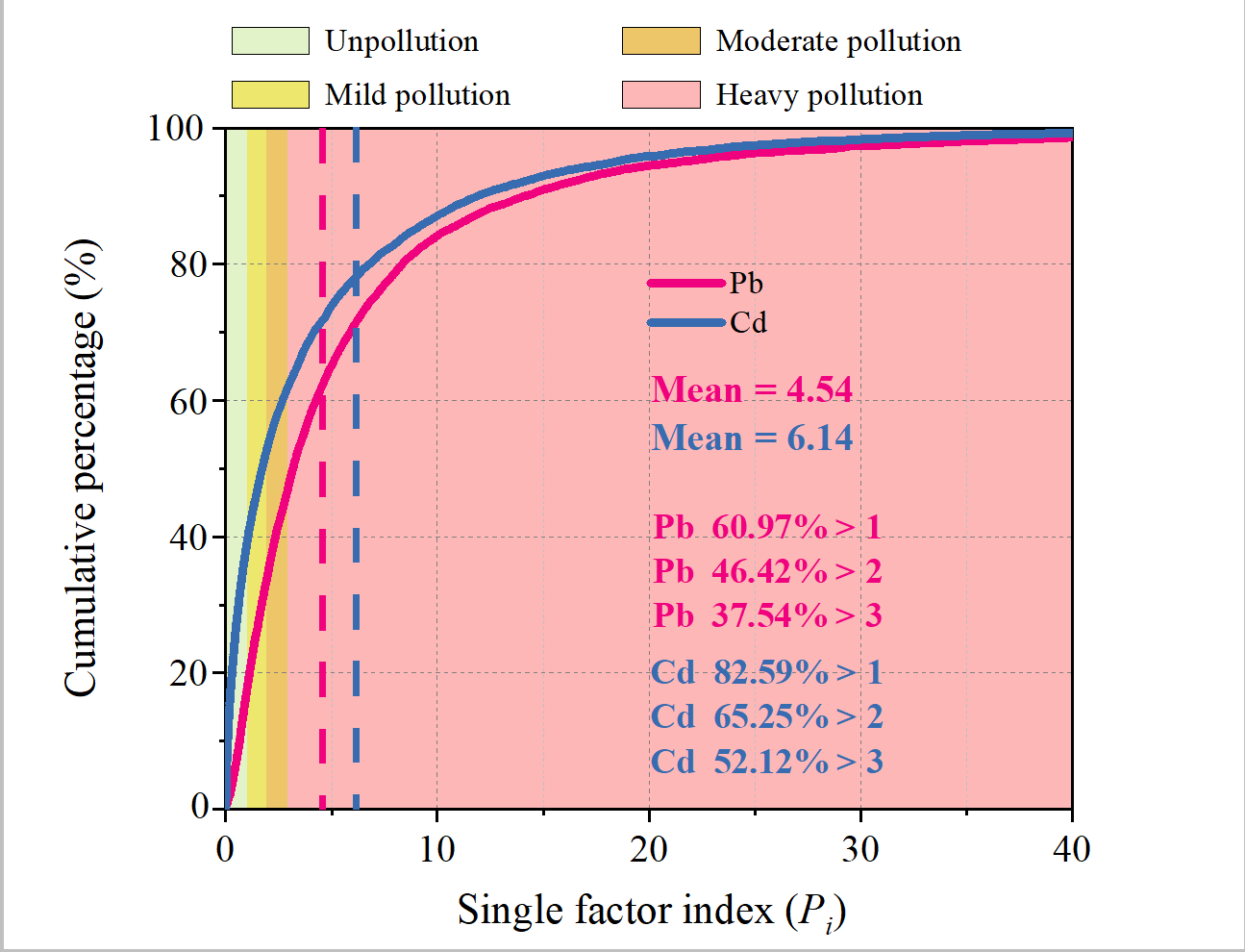


S4. Probabilistic risk assessment of heavy metal content in the Pearl River Basin

**Table**

S1:

Table S1 shows the hyperparameters of the comparison models.

S4 Comparison of model training hyperparameters.

| Model | Hyperparameters |
| --- | --- |
| Support Vector Regression (SVR) | Kernel type: linear, polynomial, RBF  C: [0.1, 1, 10, 100]  Epsilon: [0.01, 0.1, 0.5, 1] |
| Random Forest (RF)&  Spatial Random Forest(SRF) | Number of trees: [10, 50, 100, 200]  Maximum depth: [10, 20, 30, None]  Min samples split: [2, 5, 10]  Min samples leaf: [1, 2, 4] |
| Fully Connected Neural Network | Number of hidden layers: [1, 2, 3]  Units per layer: [32, 64, 128]  Activation functions: ReLU, tanh, sigmoid  Dropout rate: [0.0, 0.2, 0.5]  Learning rate: [0.001, 0.01, 0.1] |
| Convolutional Neural Network (CNN) | Number of conv layers: [1, 2, 3]  Filter size: [3x3, 5x5]  Number of filters: [32, 64, 128]  Stride: [1, 2]  Pooling size: [2x2, 3x3] |

S2：Table S2 Descriptive Statistical Analysis of Heavy Metal Content in the Soil of the Pearl River Basin

| Heavy Metal | Average Value/mg·kg-1 | Standard Layer | Coefficient of Variation/% | Skewness | Kurtosis |
| --- | --- | --- | --- | --- | --- |
| Cd | 1.58 | 2.16 | 1.37 | 4.16 | 24.73 |
| Pb | 105.83 | 176.28 | 1.67 | 2.92 | 8.53 |
